# Proteasome inhibitors reduce thrombospondin-1 release in human dysferlin-deficient myotubes

**DOI:** 10.1186/s12891-020-03756-7

**Published:** 2020-11-27

**Authors:** Esther Fernández-Simón, Cinta Lleixà, Xavier Suarez-Calvet, Jordi Diaz-Manera, Isabel Illa, Eduard Gallardo, Noemí de Luna

**Affiliations:** 1 Neuromuscular Diseases group. Institut de Recerca Hospital de Sant Pau, Biomedical Research Institute Sant Pau (IIB Sant Pau), Universitat Autónoma de Barcelona, c/Sant Antoni Mª Claret 167, 08025 Barcelona, Spain; 2 Centro de Investigación Biomédica en Red de Enfermedades Raras (CIBERER), Valencia, Spain; 3 Department of Neurology, Neuromuscular Diseases Unit, Hospital de la Santa Creu i Sant Pau, Universitat Autònoma de Barcelona, Barcelona, Spain

**Keywords:** Dysferlin, Proteasome, Vitamin D3, Myogenin, Sarcolemma, Thrombospondin-1

## Abstract

**Background:**

Dysferlinopathies are a group of muscle disorders causing muscle weakness and absence or low levels of dysferlin, a type-II transmembrane protein and the causative gene of these dystrophies. Dysferlin is implicated in vesicle fusion, trafficking, and membrane repair. Muscle biopsy of patients with dysferlinopathy is characterized by the presence of inflammatory infiltrates. Studies in the muscle of both human and mouse models of dysferlinopathy suggest dysferlin deficient muscle plays a role in this inflammation by releasing thrombospondin-1. It has also been reported that vitamin D3 treatment enhances dysferlin expression. The ubiquitin-proteasome system recognizes and removes proteins that fail to fold or assemble properly and previous studies suggest that its inhibition could have a therapeutic effect in muscle dystrophies. Here we assessed whether inhibition of the ubiquitin proteasome system prevented degradation of dysferlin in immortalized myoblasts from a patients with two missense mutations in exon 44.

**Methods:**

To assess proteasome inhibition we treated dysferlin deficient myotubes with EB1089, a vitamin D3 analog, oprozomib and ixazomib. Western blot was performed to analyze the effect of these treatments on the recovery of dysferlin and myogenin expression. TSP-1 was quantified using the enzyme-linked immunosorbent assay to analyze the effect of these drugs on its release. A membrane repair assay was designed to assess the ability of treated myotubes to recover after membrane injury and fusion index was also measured with the different treatments. Data were analyzed using a one-way ANOVA test followed by Tukey post hoc test and analysis of variance. A *p* ≤ 0.05 was considered statistically significant.

**Results:**

Treatment with proteasome inhibitors and EB1089 resulted in a trend towards an increase in dysferlin and myogenin expression. Furthermore, EB1089 and proteasome inhibitors reduced the release of TSP-1 in myotubes. However, no effect was observed on the repair of muscle membrane after injury.

**Conclusions:**

Our findings indicate that the ubiquitin-proteasome system might not be the main mechanism of mutant dysferlin degradation. However, its inhibition could help to improve muscle inflammation by reducing TSP-1 release.

**Supplementary Information:**

The online version contains supplementary material available at 10.1186/s12891-020-03756-7.

## Background

Mutations in the dysferlin gene (*DYSF*) that lead to absence or marked reduction of the protein are the cause of dysferlinopathy [1]. The disease is an autosomal recessive muscle disorder and has several phenotypes, including limb girdle 2B [2], Miyoshi myopathy [3] and distal anterior compartment myopathy [4]. Progression of the disease is characterized by muscle weakness and atrophy, and muscle biopsy shows mainly necrosis of muscle fibers and inflammatory infiltrates. Although several genetic and pharmacologic treatments have been tested [5, 6], no curative treatment is yet available for dysferlinopathy [7].

*DYSF* contains 55 exons and 14 isoforms. Fifty-three percent of patients have nonsense mutations, 43% have missense mutations, and 4% have small in-frame insertions or deletions [1]. While nonsense mutations produce a complete absence of the protein, a residual expression of dysferlin is detected in primary myotubes from patients carrying missense mutations [8, 9].

Dysferlin protein is highly expressed in skeletal muscle, cardiac muscle and blood monocytes [10]. It is a type-II transmembrane protein that contains seven C2 domains (Ca^2+^-binding) involved in vesicle fusion, trafficking, membrane repair and regulation of calcium homeostasis [11, 12]. However, it is also associated with other processes, including intracellular signaling and myoblast differentiation [13]. Studies using primary cultures of human skeletal muscle show that dysferlin and myogenin may share a pathway involved in differentiation of skeletal muscle “in vitro” as dysferlin-deficient (dysf mut/mut) myotubes have reduced expression of myogenin and are poorly differentiated [14]. It has been reported that thrombospondin-1 (TSP-1) expression is increased in dysf mut/mut myotubes and may play a role in the inflammation observed in muscle biopsies of patients with dysferlin myopathy [15]. In fact, it has been shown that serum TSP-1 levels correlate with macrophage inflammation and muscle damage in dysferlin-deficient BlaJ mice [16].

The ubiquitin-proteasome system (UPS) is the major proteolytic pathway as more than 80% of cellular proteins are degraded therein [17]. The UPS is an ATP-dependent system that removes unfolded or misfolded proteins [18]. It interacts only with proteins labelled with a polyubiquitin chain [18]. Proteins bind proteasome through the α-rings of the 20S subunit and then pass through the β-rings, where they are degraded by the proteasome particles: chymotrypsin-like (CT-L), caspase-like (C-L), and trypsin-like (T-L) proteolytic sites [19].

Previous studies have shown that proteasome inhibitors could have a therapeutic implication in muscle dystrophies. It has been reported that the proteasome inhibitor MG-132 rescued the expression of the dystrophin-glycoprotein complex (DGC) in mouse models of Duchenne muscular dystrophy and in skeletal muscle cultures from patients with Duchenne and Becker muscular dystrophy [20–22]. Bortezomib, another proteasome inhibitor, promoted the expression and membrane localization of dystrophin and dystrophin-associated proteins in *mdx* mice, but the drug had only a modest effect in myoblasts from patients with DMD [22]. Since patients carrying missense mutations in *DYSF* have residual expression of dysferlin, prevention of its degradation by the UPS could be used as a therapeutic approach.

Apart from avoiding protein degradation, other studies have focused on increasing dysferlin expression by activating gene expression. In peripheral blood monocytes (PBM) and myotubes of dysferlinopathy carriers bearing one mutation in *DYSF*, dysferlin expression is increased after vitamin D3 treatment [8]. Vitamin D3 binds to the vitamin D3 response element (VDRE) present in the dysferlin promoter and enhances dysferlin expression. EB1089 is an analogue of vitamin D3 that shows higher efficacy and less hypercalcemic activity [23], but its effect on dysferlin expression has not been studied.

As there are no effective treatments for dysferlinopathies, we assessed whether inhibition of the ubiquitin proteasome system could prevent degradation of dysferlin in immortalized myoblasts from a patient carrying two missense mutations. We used oprozomib and ixazomib, 2 s generation proteasome inhibitors that have reduced toxicity and off-targets, increasing effectiveness. We also studied the role of EB1089 in the expression of dysferlin, alone or in combination with proteasome inhibitor. We evaluated the effect of these treatments by studying the expression of myogenin, the ability of myotubes to repair the sarcolemma after injury and the release of TSP-1.

## Material and methods

### Samples

In all experiments we used immortalized myoblasts from a dysferlinopathy patient with homozygous missense mutations (c.4882G > A/p.G1628R) in exon 44 of *DYSF* and from a healthy control. This patient corresponds to number 16 in our previous work and was described showing some degree of dysferlin expression in sarcoplasm [24]. These cells present a residual expression of dysferlin of about 1–2% compared to WT myotubes. These cells were kindly provided by Dr. Mouly [25]. Myoblasts were expanded using skeletal muscle medium (SMM; Promocell, Heidelberg, Germany) until confluence was achieved. Media was then changed to differentiation medium (75% Dulbecco’s Modified Eagle’s Medium and 25% M199, supplemented with 2% FBS (Lonza, Basel, Switzerland), 10 μg/ml insulin (Sigma-Aldrich, St Louis, MO, USA), 2 mmol/l glutamine (Lonza) and penicillin-streptomycin (Lonza)) for 7 to 9 days. Differentiated myotubes were treated with vitamin D3 or EB1089 (Sigma Aldrich) at 100 nM, ixazomib (Selleckchem, Munich, Germany) (25 nM, 50 nM and 100 nM) and oprozomib (ONX 0912)(Selleckchem) (10 nM, 50 nM and 100 nM). Ixazomib and oprozomib were combined with EB1089 at 8 h and 24 h, respectively.

### Western blot

Cell pellets corresponding to each condition were lysed in RIPA buffer (Sigma-Aldrich) containing a protease and phosphatase inhibitor cocktail (Roche, Basel, Switzerland). Lysates were centrifuged at 4 °C at 13000 x *g* for 20 min and supernatants were stored at − 80 °C. Protein concentrations were determined using Pierce™ BCA Protein Assay (Thermo Fisher Scientific, Waltham, MA, USA). Thirty micrograms of protein were resolved in a 10% sodium dodecyl sulfate (SDS) polyacrylamide gel and transferred to nitrocellulose membranes. Unspecific binding sites were blocked by incubation for 1 h in casein diluted 1:1 in tris-buffered saline (TBS). Blots were incubated overnight with the primary mouse monoclonal antibodies anti-dysferlin (NCL-Hamlet, Novocastra, Newcastle, UK) and anti-myogenin (5FD clone) (Santa Cruz Biotechnology, Dallas, TX). To normalize the results, mouse anti-desmin (Novocastra) was added simultaneously with the primary antibody. The secondary antibody for anti-dysferlin was a goat anti-mouse labeled with IR-Dye 800 (Li-Cor, Lincoln, Nebraska, USA). When the anti-myogenin antibody was used as a primary antibody, biotin-labeled horse anti-mouse (Jackson ImmunoResearch, Ely, UK) was used as a secondary antibody. Secondary antibodies were incubated for 1 h at room temperature. Membranes incubated with biotinylated secondary antibody were washed and incubated for 1 h with IRDye-680-labeled streptavidin (Li-Cor). After extensive washing, the immunoreactive bands were visualized using the Odyssey Infrared ImagingSystem (Li-Cor). The amount of protein was quantified using Image Studio Lite software (Li-Cor). Desmin expression was used as a loading control. As a reference value we used dysferlin or myogenin expression of the healthy control. Protein expression in the remaining conditions was quantified over the expression in WT myotubes and expressed as fold-change.

### Assessment of proteasome-like activity

Cells were seeded in a 96- well black plate (Sarstedt, Nümbrecht, Germany) at 5000 cells/well in 3 replicates until confluence. The media was then changed to differentiation medium. Cells were treated with the corresponding drug for the indicated time. The CT-L, C-T and T-L activity was assayed by chemiluminiscence using the Proteasome-Glo™ 3-substrate System cell based assay (Promega, Madison, WI, USA) and the plate was read using Victor 3v Multilabel Plate Reader (Perkin Elmer, Waltham, MA, USA).

### Thrombospondin-1 Enzyme-Linked Immunosorbent Assay (ELISA)

Immortalized myoblasts from the dysferlinopathy patient were seeded at 5000 cells/cm^2^ and expanded until confluence. The media was then changed to differentiation medium to form myotubes. After treatment with proteasome inhibitors together with or without EB1089, we removed the media and added 1 ml of basal DMEM (Lonza) to the culture for 24 h. Cell culture supernatants were concentrated using Amicon Ultra Centrifugal Filters 100 kb (Merck Millipore, Darmstadt, Germany). TSP-1 present in the culture media was detected using the human TSP-1 Immunoassay (Quanti-Kine ELISA, R&D Systems, Minneapolis, MN), following the manufacturer’s instructions. The detection limit of the assay was 0.355 ng/mL.

### Membrane repair assay

Immortalized myoblasts from the dysferlinopathy patient and the healthy control were grown until confluence in chambered wells and then differentiated to myotubes. The assay was performed as recently described in human muscle primary cultures [26]. SDS is an anionic surfactant that causes the leakage of intracellular components due to its ability to affect membranes [27]. Membrane injury was induced by detergent treatment as follows: after the cell cultures were washed with Hank’s Balanced Salt Solution (HBSS) (Lonza), the injury solution (HBSS with 0.12 mM or 0.25 mM SDS (Sigma-Aldrich)) was applied for 2 min. Following exposure to injury solution, cells were washed in HBSS and then incubated in recovery solution consisting of proliferation media for 90 s and 10 min. The injury and recovery steps were performed at 37 °C. Cells with injured permeable membranes were identified by exposure to propidium iodide (PI) (Sigma-Aldrich) (20 μg/mL in HBSS). If cell membrane is not repaired, PI enters the cell and penetrates the nucleus wherein it intercalates with DNA and emits fluorescence. After 2 min of exposure to the dye, cells were washed with HBSS and fixed with 4% paraformaldehyde in PBS. Nuclei were stained with Hoescht 33,342 (Invitrogen, Thermo-Fisher Scientific). In each experimental condition, we counted the total number of nuclei and the PI positive nuclei using Fiji software [28].

### Fusion index

Once membrane repair assay was performed, myotubes were permeabilized with ethanol for 5 min at room temperature. Samples were then blocked with the UltraCruz Blocking Reagent (Santa Cruz Biotechnology, Dallas, TX) for 1 h at RT. Myotubes were stained using a mouse anti-myosin heavy chain (MyHC) antibody (MF-20)(Bio-Rad, Hercules, CA, USA) for 1 h at RT and then the goat anti-mouse secondary antibody Alexa Fluor 594 (ThermoFisher, Wal- tham, MA) was used as a secondary antibody for 1 h at RT. Following the incubation with the secondary antibody, nuclei were stained with Hoescht 3342 (Invitrogen, Thermo-Fisher Scientific). Images were acquired and analysed using Fiji software.

The fusion index was determined as the percentage of nuclei included in MF-20-expressing myotubes (containing at least 3 nuclei) divided by the total number of nuclei.

### Statistical analysis

Multiple comparisons were analyzed using a one-way ANOVA test followed by Tukey post hoc test. When statistical comparisons were performed between two groups, the nonparametric Mann-Whitney test was used. GraphPad Prism 5.0 software was used (LaJolla, CA, USA) for graphic representation.

## Results

### EB1089 and vitamin D3 treatment

**Fig. 1:**
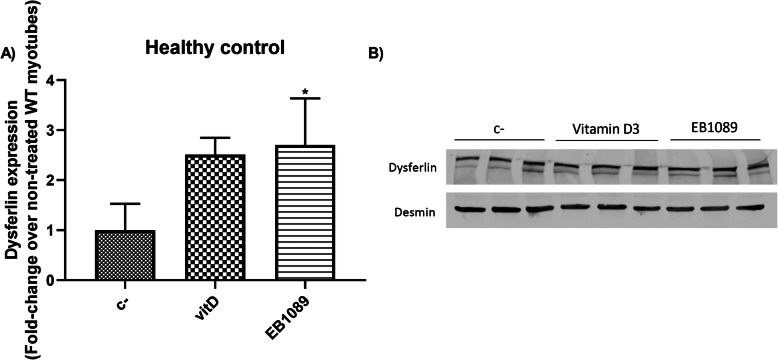
Effect of Vitamin D3 and EB1089 on healthy control myotubes. **a** Quantification of WB bands of untreated wild-type myotubes (C- ), Vitamin D3 and EB1089 treated wild-type myotubes for 24 h. Vitamin D3 treatment produced an increase in dysferlin expression compared to non-treated myotubes. However, only EB1089 showed a significant increase compared to non-treated myotubes. **b** Representative WB of dysferlin with vitamin D3 and EB1089 treatment. Desmin was used as a loading control. Data are represented as mean of 3 replicates ± standard deviation. Fold-change is calculated over the results of dysferlin expression in non-treated WT myotubes. Results were statistically analyzed using one-way Anova followed by Tukey post hoc test. Statistical significance was set at *p* < 0.05. *p < 0.05

Vitamin D3 and EB1089 were added to healthy control myotubes for 24 h at 100 nM in order to assess their effect on dysferlin expression. Both Vitamin D3 and EB1089 increased dysferlin expression. However, only treatment with EB1089 showed statistically significant differences compared to non-treated myotubes (Fig. 1). Therefore, from this point on, we decided to use EB1089 in combination with proteasome inhibitors to increase dysferlin expression.

### Proteasome inhibition

We analyzed the time points at which ixazomib and oprozomib produced the highest increase in dysferlin expression in dysf mut/mut myotubes. The effect of ixazomib was highest at 8 h and the effect of oprozomib was highest at 24 h (data not shown). We also analyzed whether the addition of EB1089 to the proteasome inhibitors had an effect on dysferlin expression at the same time points.

**Fig. 2:**
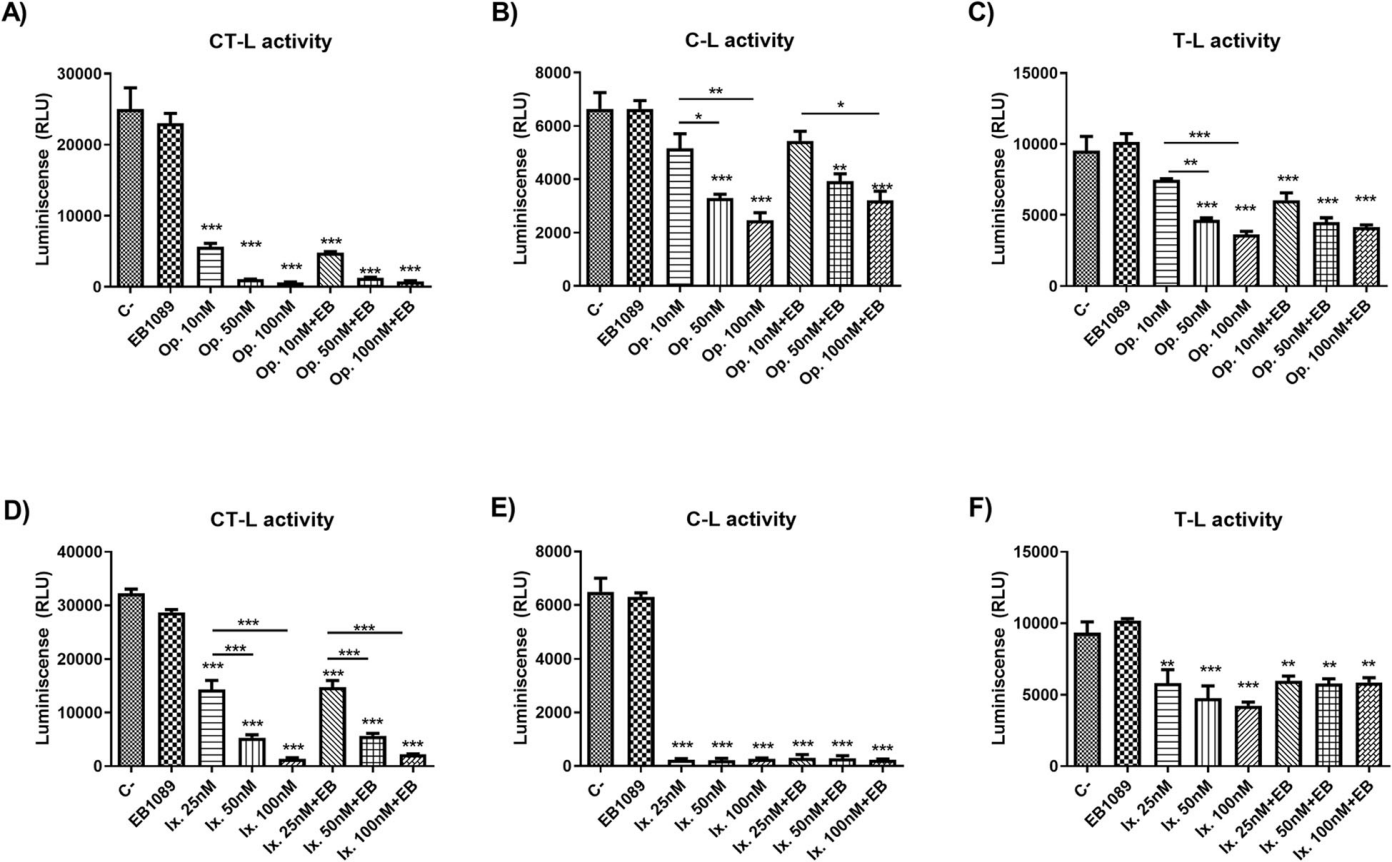
Proteasome inhibition profile. The three distinct ATPase-independent proteolytic activities (CT-L, C-L and T-L) of the proteasome were assayed using a chemioluminiscence-based method. Dysf mut/mut myotubes were pretreated with oprozomib and EB1089 for 24 h. Oprozomib treatment presented inhibition at: **a** CT-L at all concentrations tested **b**) C-L, and **c**) T-L activity also were inhibited by oprozomib, but only at high doses (50 nM and 100 nM). Pre-treatment with ixazomib and EB1089 for 8 h was also assayed and **d**) CT-L, **e**) C-L and **f**) T-L activity were inhibited at all doses tested with significant differences. Data are represented as mean of 3 replicates ± standard deviation. Results were statistically analyzed using one-way Anova followed by Tukey post hoc test. Statistical significance was set at *p* < 0.05. *p < 0.05; **p < 0.01; ***p < 0.0001

Treatment of dysf mut/mut myotubes with 10 nM oprozomib for 24 h showed high selectivity towards the CT-L active site of the proteasome. However, at higher doses (50 nM and 100 nM) oprozomib also inhibited C-L and T-L activity significantly (Fig. 2a-c).

Ixazomib treatment inhibited all 3 proteasome subunits (Fig. 2d-f).

When EB1089 was added alone to the culture, no proteasome inhibition in any subunit was found. Moreover, when it was combined with ixazomib and oprozomib, no effect was observed in the inhibition of each subunit of the proteasome.

### Dysferlin and myogenin expression

We analyzed the expression of dysferlin and myogenin in dysf mut/mut myotubes treated with EB1089, oprozomib and ixazomib.

**Fig. 3:**
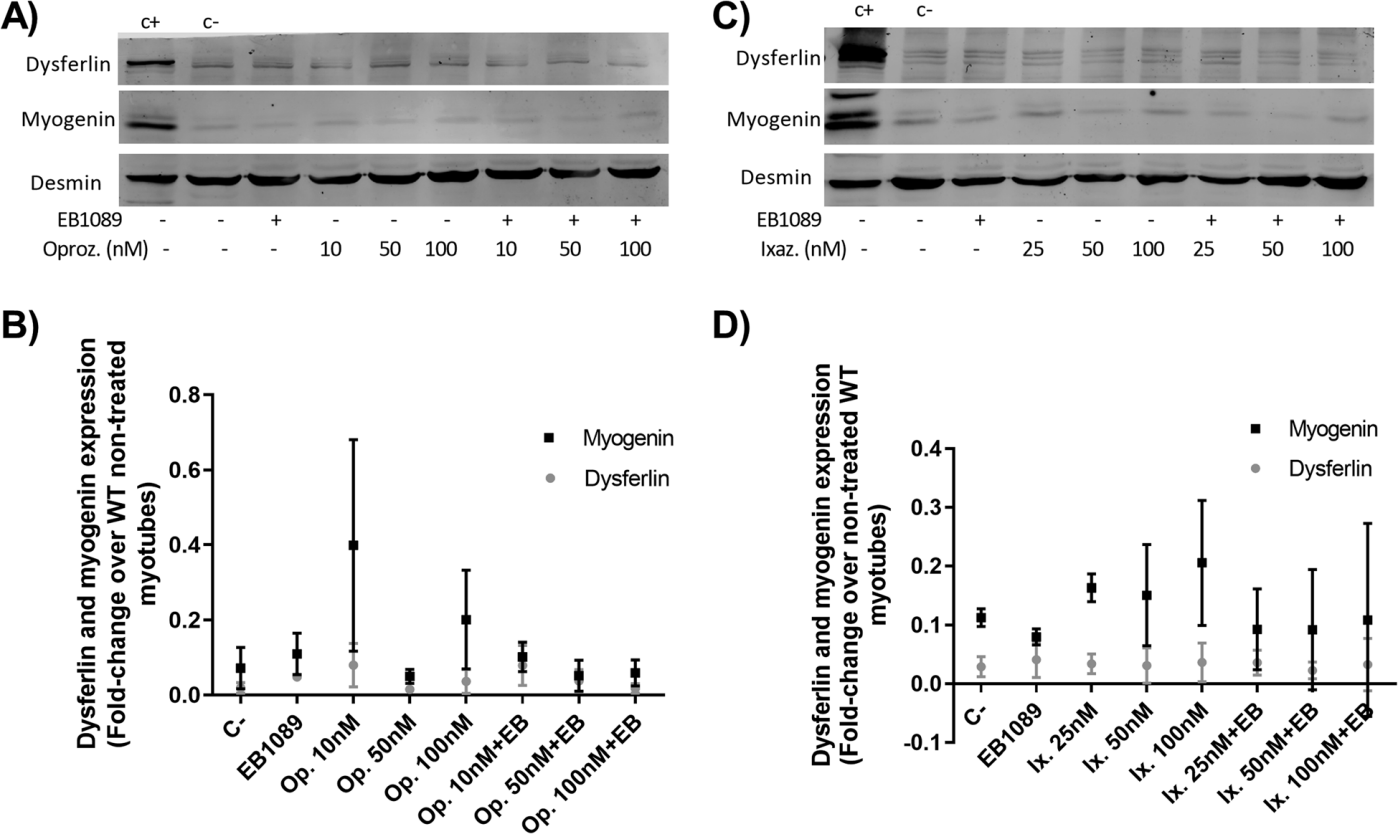
Dysferlin and myogenin expression in dysf mut/mut muscle cells. **a** Representative WB of myogenin and dysferlin with oprozomib and EB1089 treatment. C+: untreated wild-type myotubes, (C-) untreated dysf mut/mut myotubes. Desmin was used as loading control. **b** Quantification of WB bands of dysferlin or myogenin expression. The highest expression of dysferlin and myogenin was reached using oprozomib at 10 nM. **c** Representative WB of myogenin and dysferlin with ixazomib and EB1089. **d** Quantification of WB showed that 100 nM of ixazomib presented the highest expression of dysferlin and myogenin. Data are represented as the mean of 3 replicates ± standard deviation. The fold-change is calculated over the results of dysferlin expression in WT non-treated myotubes. Results were statistically analyzed using one-way Anova followed by Tukey post hoc test. Statistical significance was set at *p* < 0.05

Treatment with oprozomib at 24 h showed a trend towards an increase in myogenin expression (Fig. 3a-b). When EB1089 was added together with oprozomib, myogenin presented a trend towards decreased expression compared to oprozomib treatment alone. Dysferlin presented a trend towards increased expression with 10 nM of oprozomib with and without EB1089. No statistical significances were observed in any experimental conditions.

Treatment with ixazomib (Fig. 3c-d) showed no significant increase in the expression of dysferlin. Myogenin levels showed a trend towards increased expression after ixazomib treatment, being 100 nM the concentration at which the myogenin expression was the highest. The addition of EB1089 together with ixazomib presented a trend towards decreased expression of myogenin compared to ixazomib treatment alone, but the results did not reach statistical significance.

### Expression of TSP-1

**Fig. 4:**
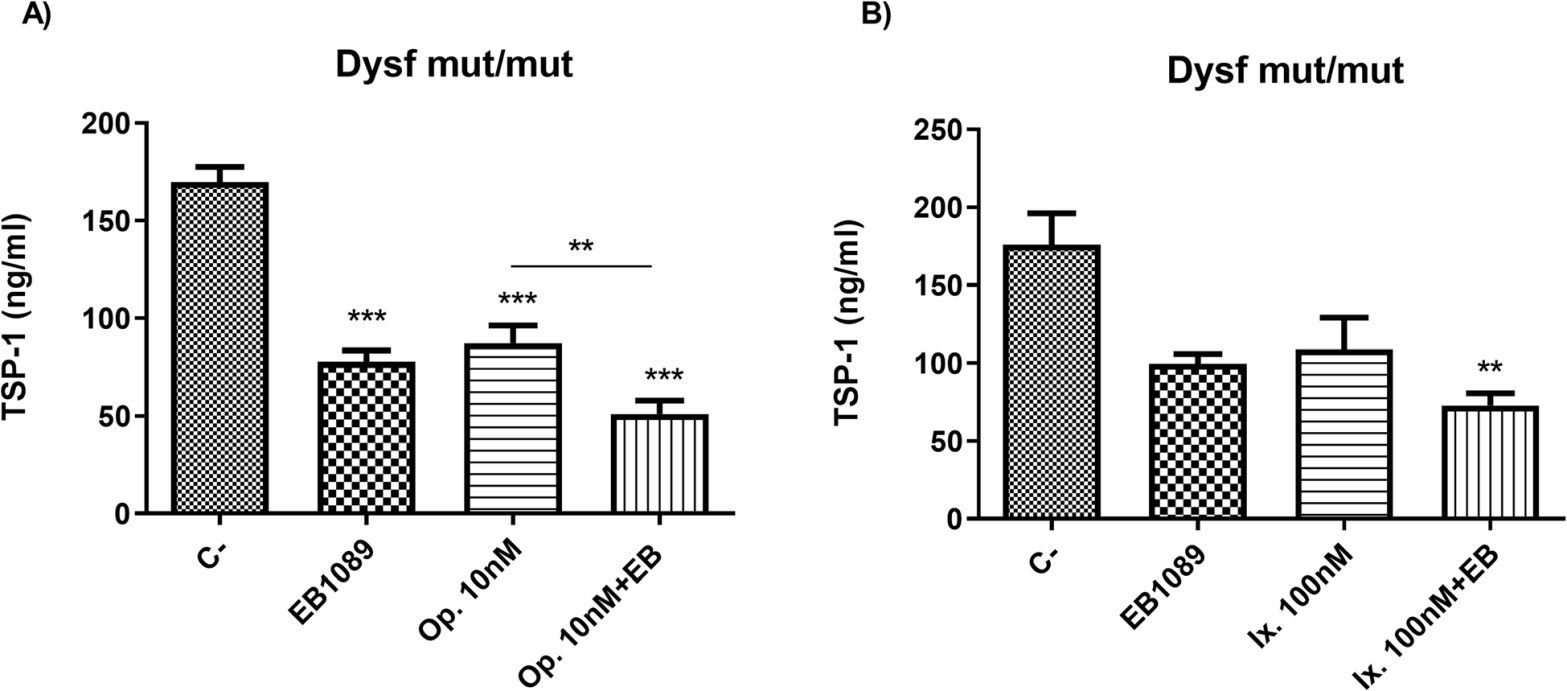
TSP-1 expression by dysf mut/mut myotubes treated with oprozomib, ixazomib and EB1089. **a** TSP-1 expression with treatment of oprozomib at 10 nM and EB1089 100 nM was significantly decreased compared to C- (non-treated dysf mut/mut myotubes), and the combination of both treatments was also significantly decreased compared to oprozomib treatment alone. **b** There were no significant differences in TSP-1 levels between ixazomib and EB1089 compared to non-treated myotubes. However, the combination of both treatments showed a significant reduction compared to non-treated myotubes. Data are represented as mean of 5 replicates ± standard deviation. Results were statistically analyzed using one-way Anova followed by Tukey post hoc test. Statistical significance was set at *p* < 0.05. **p < 0.01; ***p < 0.0001

We evaluated the secretion of TSP-1 by dysf mut/mut myotubes treated with EB1089, oprozomib and ixazomib. Treatment with EB1089 significantly reduced TSP-1 release at 8 h. TSP-1 levels also decreased significantly in dysf mut/mut cells treated with oprozomib and EB1089 separately (Fig. 4a). TSP-1 levels decreased even more significantly when cells were treated with the combination of EB1089 and oprozomib.

Ixazomib treatment significantly decreased TSP-1 only when combined with EB1089 (Fig. 4b). However, although EB1089 and ixazomib alone did not produce significant changes in TSP-1 expression, expression was lower than in untreated myotubes.

Raw western-blot data are available in Supplementary Figure 1.

### Sarcolemma repair assay

**Fig. 5:**
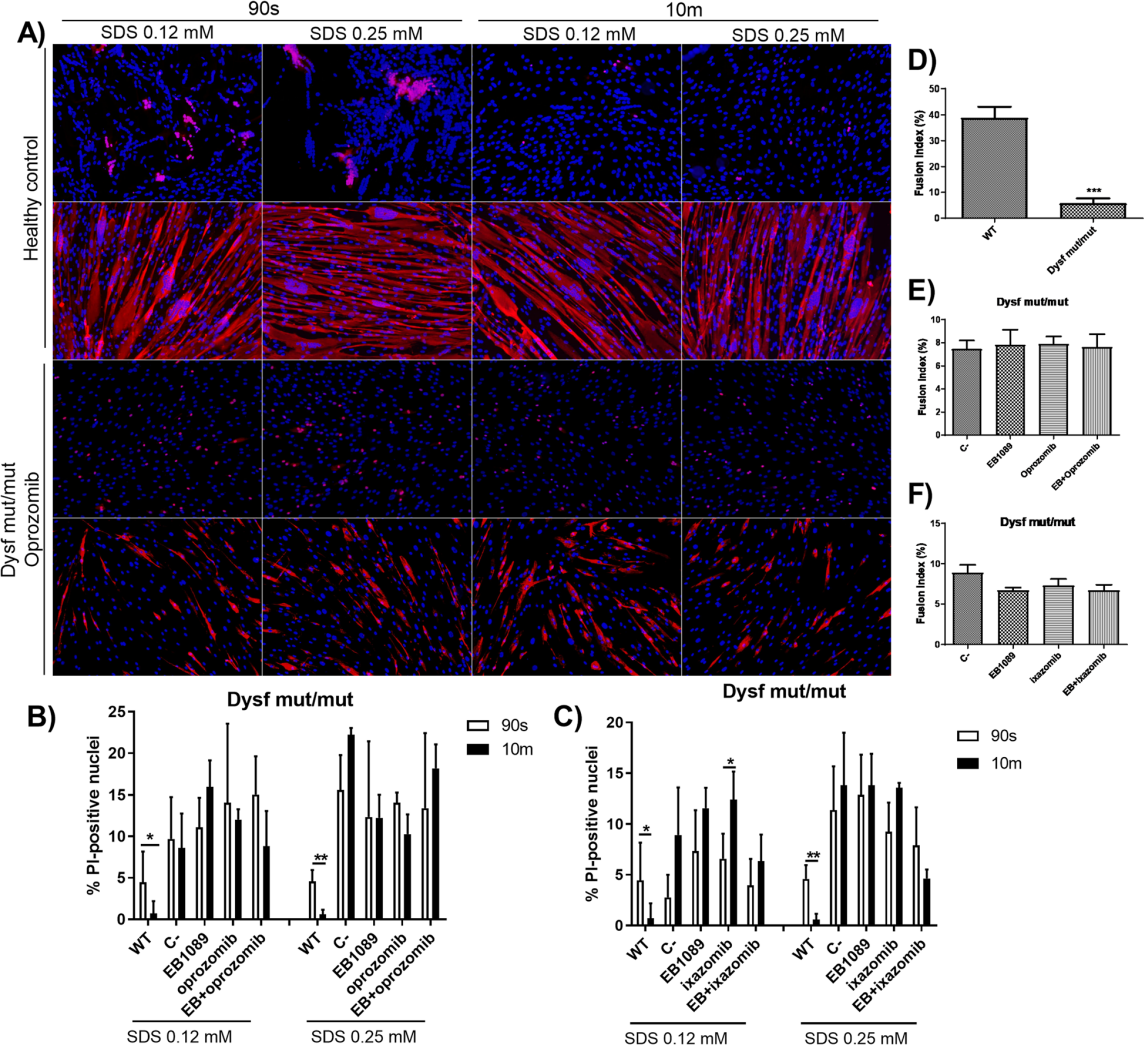
Membrane repair and fusion index using oprozomib, ixazomib and EB1089 treatment. **a** Representative images of PI-positive nuclei and MyHC positive myotubes after SDS treatment at 90 s and at 10 min. The first 2 rows correspond to healthy myotubes and the last 2 rows correspond to dysf mut/mut myotubes treated with oprozomib. **b** Quantification of IP-positive nuclei in myotubes from healthy controls and dysf mut/mut myotubes treated with EB1089, oprozomib, and the combination of oprozomib 10 nM with EB1089at at 24 h. **c** Quantification of IP-positive nuclei in myotubes from healthy controls and dysf mut/mut myotubes treated with EB1089, ixazomib, and the combination of ixazomib 100 nM with EB1089at at 8 h. Differences were not observed with any treatment. **d** Quantification of fusion index showed a statistically significant decrease of MyHC positive myotubes compared to healthy myotubes. **e** Fusion index in dysf mut/mut myotubes treated with EB1089, oprozomib at 10 nM and the combination of oprozomib 10 nM with EB1089 at 8 h did not show any difference compared to non-treated myotubes. **f** Fusion index in dysf mut/mut myotubes treated with EB1089, ixazomib at 100 nM and the combination of ixazomib 100 nM with EB1089 at 24 h did not show any difference compared to non-treated myotubes. Data are represented as mean of 3 replicates ± standard deviation. Results were statistically analyzed using Mann-Whitney test to compare two groups and one-way Anova followed by Tukey post hoc test for multiple comparisons. Statistical significance was set at *p* < 0.05. *p < 0.05; **p < 0.01; ***p < 0.0001

Considering that dysferlin is involved in membrane repair after injury, we analyzed the ability of dysf mut/mut myotubes, treated with proteasome inhibitors and EB1089, to repair the sarcolemma after injury. We found that wild-type myotubes repaired the membrane at both low and high concentration of SDS (0.12 mM SDS and at 0.25 mM SDS), showed by PI exclusion, 10 min after injury (Fig. 5a). However, dysf mut/mut myotubes did not present any sarcolemma repair in any condition tested (Fig. 5a-c).

### Fusion index

Fusion index results showed that dysf mut/mut myotubes presented a statistically significant lower fusion index compared to wild-type myotubes (Fig. 5e). Ixazomib, oporozomib and EB1089 did not show any effect on the fusion index of dysf mut/mut myotubes (Fig. 5f-g).

## Discussion

The aim of this study was to test if blockade of proteasome could restore dysferlin expression and consequently rescue the muscle functions that are impaired when dysferlin is missing. It has been reported that dysferlin deficient myoblasts display reduced levels of myogenin and consequently fusion index is reduced [14]. Muscles biopsies of dysferlinopathy patients are characterized by the presence of abundant inflammatory infiltrates [29], TSP-1 seems to have a prominent role in muscle inflammation in this pathology [15]. Finally, dysferlin is involved in sarcolemma repair [26, 30].

Treatment with oprozomib, ixazomib and EB1089 in myotubes from a dysferlinopathy patient promoted a trend towards increased expression of dysferlin and myogenin, but this low increase did not translate into higher sarcolemmal repair neither higher fusion index than that in untreated myotubes. However, TSP-1 release from myotubes decreased after treatment.

Western blot analysis showed a trend towards increased expression of dysferlin and myogenin when oprozomib and EB1089 were added to the culture. Ixazomib produced a trend towards increased expression of myogenin but not dysferlin. These results are in agreement with a previous work by Fujita E et al. They found that mutant-dysferlin was mainly degraded by autophagy while wild type-dysferlin was degraded by the ubiquitin-proteasome system, although they used a proteasome inhibitor different from those used in this study [31]. The small increase in dysferlin expression that we observed after treating dysf mut/mut myotubes with our different ubiquitin-proteasome inhibitors confirms that this system is not the main pathway to recycle mutated dysferlin.

The other treatment that we studied, the analogue of Vitamin D3, EB1089, produced a trend towards increased expression of dysferlin. In other studies, authors found that Vitamin D3 treatment had no effect on dysferlin expression in myotubes from dysferlinopathy patients but increased dysferlin expression in muscle cultures from carriers of one mutation in *DYSF* [8]. Although EB1089 is 50–200 times more potent than vitamin D3 [23], when added to cultured myotubes from a dysferlinopathy patient it only produces a trend towards an increase in dysferlin expression. Vitamin D3 acts through the Vitamin D receptor and p38 MAPK and participates in differentiation events in skeletal muscle cells [32]. Whether this effect is dependent on dysferlin expression remains to be elucidated. Moreover, EB1089 also presented a trend towards decreased expression of myogenin when was combined with proteasome inhibitors compared to the results of treatment with a proteasome inhibitor alone. Although results were not statistically significant, this negative effect could be explained by a negative regulation of myogenin after EB1089 treatment. In previous studies, authors showed that Vitamin D3 downregulates the expression of myogenin both in vitro and in vivo [33–35]*.* These previous findings suggest that negative vitamin D response elements may be present in the promoter region of myogenin, however those have not been identified [36].

We also analyzed the expression of TSP-1 in cell cultures after treatment with proteasome inhibitors. TSP-1 is secreted in response to inflammation and multiple factors modulate its release. It is expressed by endothelial cells, fibroblasts, neutrophils, macrophages, T cells, and myotubes [37]. We observed a significant decrease in TSP-1 release when dysf mut/mut myotubes were exposed to EB1089, oprozomib and the combination of oprozomib or ixazomib and EB1089. The decrease in TSP-1 levels could be the consequence of the trend towards an increased expression of dysferlin in these conditions. However, TSP-1 levels were significantly lower when a combination of oprozomib and EB1089 was added to the culture than when oprozomib was added alone, suggesting that proteasome inhibitors and EB1089 act independently of dysferlin expression to reduce TSP-1 release.

The reduced expression of TSP-1 observed after treatment with EB1089 could be explained by mechanisms independent of dysferlin expression. Amarasekera AT et al. suggested that vitamin D supplementation reduces TSP-1 levels in healthy individuals [38]. Moreover, it has been studied that calcitrol, a vitamin D metabolite, downregulates TSP-1 via the activation of the mitogen activated protein kinase (MAPK) [39]. Therefore, EB1089 treatment could activate the MAPK pathway leading to downregulation of TSP-1. This activation could constitute an alternative mechanism to reduce TSP-1 levels that is not linked to dysferlin expression.

The effect of proteasome inhibitors on TSP-1 could also be explained by mechanisms non-related to dysferlin expression, such as the inhibition of canonical NF-κB signaling. NF-κB forms a dimer with IκB, an inhibitory protein, which keeps the complex inactive in the cytoplasm. When signals activate this pathway, IκB is phosphorylated and degraded by the proteasome, releasing the NF-κB to the nucleus where it regulates the transcription of genes involved in the inflammatory response. Proteasome inhibitors seem to decrease the nuclear levels of NF-κB by avoiding the degradation of IκB, hence decreasing NF-κB activity [40]. In effect, in dysferlinopathy patients, damage-associated molecular patterns (DAMPs) are released from damaged muscle fibers, triggering inflammatory responses via activation of NF-κB [41]. We suggest that decreased NF-κB activity by proteasome inhibitors could be another mechanism involved in TSP-1 reduction and thereby reduce the inflammatory response.

We did not observe any sarcolemma repair when proteasome inhibitors and EB1089 were added to the culture. We have previously reported that dysferlin mut/mut cells fuse less efficiently than wild-type myoblasts. We also demonstrated that dysferlin mut/mut myotubes showed a reduced expression of myogenin in the nucleus [14]. The experiments performed in the present study confirm that fusion of myoblasts is impaired in dysferlin mut/mut. Lack of dysferlin impaires sarcolemmal repair both in myoblasts and myotubes therefore, we believe that reduced fusion of myoblasts has no effect on the membrane repair experiments. The trend towards increased expression of dysferlin after treatment was not strong enough to restore sarcolemma repair of damaged myotubes.

## Conclusions

In conclusion, we did not observe a significant increase of dysferlin or myogenin in myotubes from a dysferlinopathy patient when treated with EB1089, oprozomib and ixazomib. However, we did observe a significant reduction in TSP-1 levels. Targeting TSP-1 may constitute a therapeutic approach in patients carrying mutations affecting proper folding of dysferlin since it would reduce inflammation and avoid the undesirable side-effects reported in patients with dysferlinopathy treated with prednisone.

## Supplementary Information

**Additional file 1:** **Supplementary Figure 1.**` Dysferlin and myogenin expression in dysf mut/mut muscle cells. A) Representative full blot of dysferlin with vitamin D3 and EB1089 treatment. Desmin was used as a loading control. B) Representative full blot of myogenin and dysferlin with oprozomib and EB1089 treatment. C) Representative full blot of myogenin and dysferlin with ixazomib and EB1089 treatment.

## Data Availability

The datasets used and/or analyzed during the current study are available from the corresponding author on reasonable request.

## Abbreviations

DYSF: Dysferlin
C2: Ca^2+^-binding
Dysf mut/mut: Dysferlin deficient
TSP-1: Thrombospondin 1
UPS: Ubiquitin-proteasome system
C-L: Caspase-like
T-L: Trypsin-like
CT-L: Chemotrypsin-like
DGC: Dystrophin-glycoprotein complex
PBM: Peripheral blood monocytes
VDRE: Vitamin D3 response element
PI: Propidium iodide
DAMP: Damage-associated molecular pattern
