# Supplementary material for: Proteasome inhibitors reduce thrombospondin-1 release in human dysferlin-deficient myotubes
Source: BMC Musculoskelet Disord. 2020 Nov 27;21:784. doi: 10.1186/s12891-020-03756-7 (PMC7697384; doi:10.1186/s12891-020-03756-7)
Supplement: Supplementary file 1 — Additional file 1: Supplementary Figure 1.` Dysferlin and myogenin expression in dysf mut/mut muscle cells. A) Representative full blot of dysferlin with vitamin D3 and EB1089 treatment. Desmin was used as a loading control. B) Representative full blot of myogenin and dysferlin with oprozomib and EB1089 treatment. C) Representative full blot of myogenin and dysferlin with ixazomib and EB1089 treatment. [file 12891_2020_3756_MOESM1_ESM.docx]

**
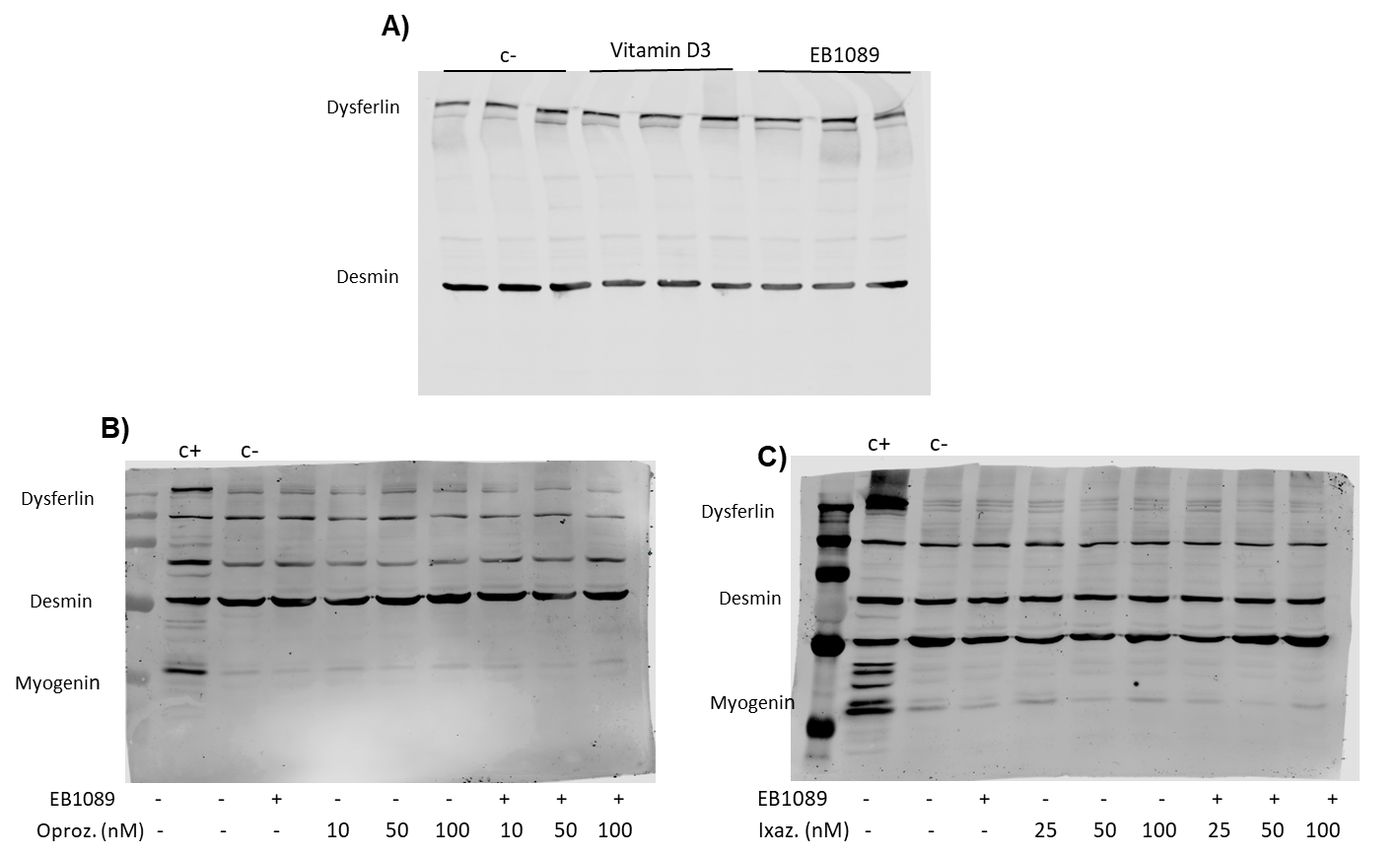
**

**Supplementary figure 1. Dysferlin and myogenin expression in dysf mut/mut** **muscle cells. A)** Representative full blot of dysferlin with vitamin D3 and EB1089 treatment. Desmin was used as a loading control. B) Representative full blot of myogenin and dysferlin with oprozomib and EB1089 treatment. C) Representative full blot of myogenin and dysferlin with ixazomib and EB1089 treatment.
